# Microcarriers Based on Glycosaminoglycan-Like Marine Exopolysaccharide for TGF-β1 Long-Term Protection

**DOI:** 10.3390/md17010065

**Published:** 2019-01-19

**Authors:** Agata Zykwinska, Mélanie Marquis, Mathilde Godin, Laëtitia Marchand, Corinne Sinquin, Catherine Garnier, Camille Jonchère, Claire Chédeville, Catherine Le Visage, Jérôme Guicheux, Sylvia Colliec-Jouault, Stéphane Cuenot

**Affiliations:** 1 Ifremer, Laboratoire Ecosystèmes Microbiens et Molécules Marines pour les Biotechnologies, F-44311 Nantes, France; Godin.Mathilde@ymail.com (M.G.); Laetitia.Marchand@ifremer.fr (L.M.); Corinne.Sinquin@ifremer.fr (C.S.); Sylvia.Colliec.Jouault@ifremer.fr (S.C.-J.); 2 INRA, UR1268 Biopolymères Interactions Assemblages, F-44300 Nantes, France; melanie.marquis@inra.fr (M.M.); catherine.garnier@inra.fr (C.G.); camille.jonchere@inra.fr (C.J.); 3 Inserm, UMR 1229, RMeS, Regenerative Medicine and Skeleton, Université de Nantes, ONIRIS, F-44042 Nantes, France; claire.chedeville@univ-nantes.fr (C.C.); catherine.levisage@inserm.fr (C.L.V.); jerome.guicheux@inserm.fr (J.G.); 4 UFR Odontologie, Université de Nantes, F-44042 Nantes, France; 5 CHU Nantes, PHU 4 OTONN, F-44093 Nantes, France; 6 Institut des Matériaux Jean Rouxel (IMN), Université de Nantes-CNRS, 44322 Nantes, France; Stephane.Cuenot@cnrs-imn.fr

**Keywords:** exopolysaccharide, growth factor, microparticles, microfluidics, bioactivity

## Abstract

Articular cartilage is an avascular, non-innervated connective tissue with limited ability to regenerate. Articular degenerative processes arising from trauma, inflammation or due to aging are thus irreversible and may induce the loss of the joint function. To repair cartilaginous defects, tissue engineering approaches are under intense development. Association of cells and signalling proteins, such as growth factors, with biocompatible hydrogel matrix may lead to the regeneration of the healthy tissue. One current strategy to enhance both growth factor bioactivity and bioavailability is based on the delivery of these signalling proteins in microcarriers. In this context, the aim of the present study was to develop microcarriers by encapsulating Transforming Growth Factor-β1 (TGF-β1) into microparticles based on marine exopolysaccharide (EPS), namely GY785 EPS, for further applications in cartilage engineering. Using a capillary microfluidic approach, two microcarriers were prepared. The growth factor was either encapsulated directly within the microparticles based on slightly sulphated derivative or complexed firstly with the highly sulphated derivative before being incorporated within the microparticles. TGF-β1 release, studied under in vitro model conditions, revealed that the majority of the growth factor was retained inside the microparticles. Bioactivity of released TGF-β1 was particularly enhanced in the presence of highly sulphated derivative. It comes out from this study that GY785 EPS based microcarriers may constitute TGF-β1 reservoirs spatially retaining the growth factor for a variety of tissue engineering applications and in particular cartilage regeneration, where the growth factor needs to remain in the target location long enough to induce robust regenerative responses.

## 1. Introduction

**Figure 1:**
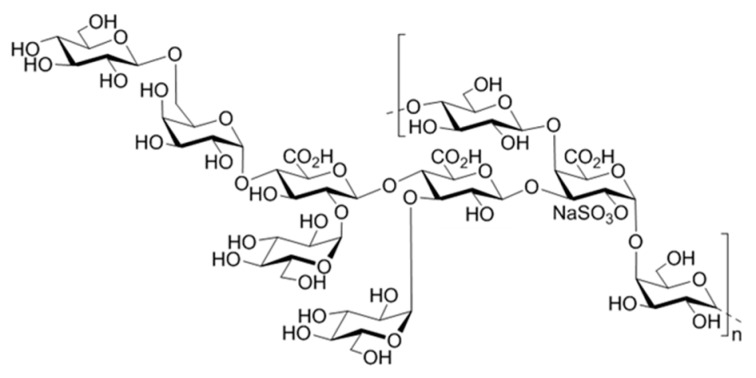
Structure of the monosulphated nonasaccharide repeating unit of the GY785 EPS.

Articular cartilage is a highly specialized tissue containing a unique cell type, the chondrocyte, able to synthesize an extracellular matrix (ECM) composed of proteoglycans, mainly aggrecan and fibrillar collagens [1,2]. The resulting aggrecan/collagen network provides the tissue its resistance to tensile and compressive strengths. However, the integrity of this hydrated pressure-resistant gel network can be altered due to aging, traumas or osteo-articular diseases. Since articular cartilage is an avascular tissue, the progenitor cells from the blood cannot migrate to reconstruct the damaged site [3]. To overcome limited intrinsic healing capacity of cartilage, several strategies have been developed for many years to support a recovery of the functional cartilaginous tissue, amongst microfracture, mosaicplasty and autologous chondrocyte implantation [3,4]. However, several barriers arising from these techniques, such as formation of transient fibrocartilaginous tissue and donor-site morbidity led to the development of a new therapeutic strategy, namely tissue engineering [4,5,6]. Tissue engineering approach is based on association of three key constituents: cells, signalling proteins (such as growth factors) and a matrix scaffold. Indeed, a pivotal feature for successful regeneration of tissue is the creation of an appropriate microenvironment supporting cell proliferation, differentiation and secretory activities. As a cell source, mesenchymal stem cells (MSC) have attracted much attention in tissue engineering due to their accessibility and their multipotency, which implies their ability to differentiate into several lineages under appropriate stimuli [5,7,8,9,10]. Several cytokines belonging to Transforming Growth Factor-β (TGF-β) superfamily, such as TGF-β1 and TGF-β3 have shown their ability to induce the chondrogenic differentiation of MSC in vitro [11,12]. The major drawback related to growth factor use is that these signalling proteins are extremely fragile with short lifetime due to their rapid enzymatic degradation. However, to reach an appropriate regenerative effect, the tissue often needs to be exposed to growth factors for relatively long timeframes. In vivo, growth factors are protected and stabilized through their non-covalent binding with the ECM components and in particular, sulphated GAG. By interacting with growth factors, GAG protect them from undesired proteolytic degradation and enhance both their local concentration up to levels required for signalling and their stability, thus facilitating the growth factor binding to their receptors [13,14,15]. Therefore, because matrix-bound growth factors remain more effective than their soluble counterparts, many delivery systems have been developed allowing both protection of growth factors from undesired degradation and their sustained release [16,17,18]. Since biocompatibility and biodegradability are the most critical parameters in tissue engineering, growth factor delivery systems based on natural polymers, mainly polysaccharides, among which alginate, chitosan, pullulan and hyaluronic acid have been engineered at macro, micro and nanoscales [19,20,21,22,23,24]. In order to enhance the growth factor binding to the matrix, polysaccharides are frequently chemically sulphated to mimic structural and functional features of highly sulphated GAG components of the ECM, such as heparan sulphate and chondroitin sulphate [22,25]. In this context, a highly sulphated low-molecular weight (LMW) derivative, namely GY785 DRS, prepared from a naturally slightly sulphated high-molecular weight (HMW) marine exopolysaccharide, GY785 EPS (Figure 1) [26,27], was shown to stimulate in vitro the chondrogenic differentiation of the human adipose-derived stromal cells (hASC) in the presence of TGF-β1 [28]. In comparison, a slightly sulphated LMW derivative, namely GY785 DR, had no effect on hASC chondrogenesis, however its presence significantly increased cell viability and proliferation. 

By taking into account these previous results, the aim of the present study was to exploit both physico-chemical (gelling) and biological (GAG-like) properties of the GY785 EPS derivatives to develop microcarriers for a long-term protection and stabilization of the encapsulated TGF-β1. To preserve its bioactivity, TGF-β1 was gently encapsulated inside two EPS-based matrices structured at micro-scale using a capillary microfluidic approach. Over other conventional emulsification techniques used for gelled microparticle generation, microfluidics offers the possibility to produce monodisperse microparticles with tailored sizes and morphologies, without using high energy and temperature conditions, which may be destructive for the bioactive molecule to be encapsulated [29,30]. Another important advantage of microfluidic is the ability to produce microstructures and, in the same time, to encapsulate the totality of the bioactive molecule, in a one-step process [31,32]. In the first instance, TGF-β1 was directly encapsulated inside gelled microparticles formed by physical cross-linking of slightly sulphated derivative, GY785 DR, mediated by calcium ions (single microcarrier, GY785 DR/TGF-β1). Furthermore, in order to additionally protect the TGF-β1, the growth factor was firstly incubated with the highly sulphated LMW derivative, GY785 DRS and the remaining complex was then encapsulated inside GY785 DR microparticles during their formation (double microcarrier, GY785 DR/DRS/TGF-β1). The release of the growth factor from both systems was followed under in vitro model conditions and the bioactivity of the released protein was assessed.

## 2. Results and Discussion

### 2.1. Gelling Properties of the Native GY785 EPS and its Derivative, GY785 DR 

**Figure 2:**
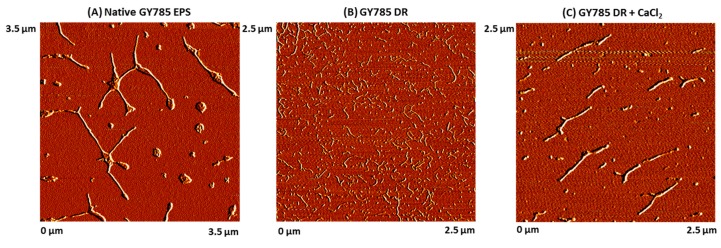
AFM images of (**A**) the native GY785 EPS (3.5 µm × 3.5 µm), (**B**) the depolymerized GY785 DR (240,000 g/mol) (2.5 µm × 2.5 µm) and (**C**) the depolymerized GY785 DR in the presence of CaCl_2_ (2.5 µm × 2.5 µm).

**Figure 3:**
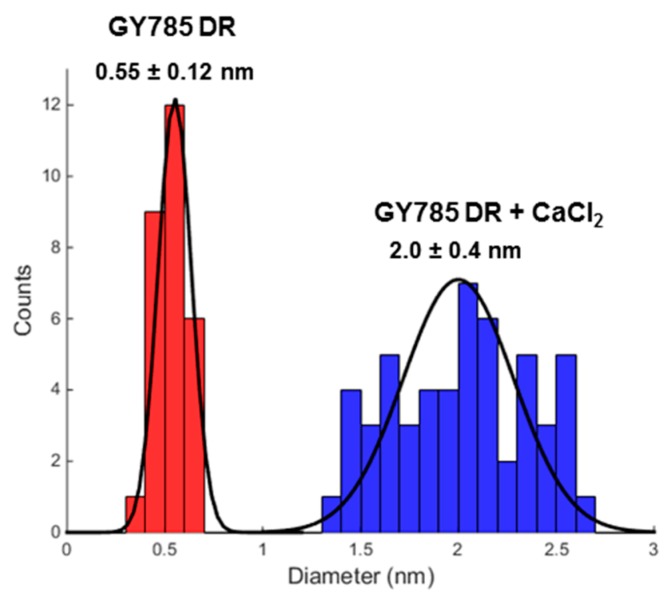
Measured diameter distribution of GY785 DR fibres before (in red) and after (in blue) calcium addition.

GY785 EPS is a highly branched naturally slightly sulphated anionic heteropolysaccharide synthesized by *Alteromonas infernus*, the deep-sea hydrothermal vent bacterium [26]. The repeating unit of this polysaccharide is constituted of nine osidic residues, which are assembled in a unique structure (Figure 1) [27]. The main chain of the polysaccharide is composed of glucose (Glc), galacturonic acid (GalA) and galactose (Gal) residues. GalA residue is substituted at *O*-2 by a sulphate group and at *O*-3 by a side chain composed of two glucuronic acids (GlcA), bearing each a terminal Glc, Gal and Glc. The presence of three consecutive uronic acids per repeating unit in the EPS structure may prompt its structuring into microparticles through physical gelation induced by divalent cations, such as Ca^2+^ as described for pectin and alginate cross-linking [33,34]. Indeed, mild conditions offered by physical gelation with the use of non-toxic reactants are particularly well-suited for bioactive molecule encapsulation. However, when mixed in a glass tube with CaCl_2_ aqueous solution, native HMW GY785 EPS (2,000,000 g/mol) was not able to gel (data not shown). It is likely that long GY785 EPS fibres observed by AFM in a dried highly-diluted sample (Figure 2A) prevented from efficient inter-chain associations and formation of stable junction zones between dissociated carboxyl groups of the polysaccharide chains and Ca^2+^ ions, as described in the “egg-box” model [33]. To favour gelation, the native HMW EPS was depolymerized to obtain its derivative, namely GY785 DR, of intermediate molar mass (240,000 g/mol). Constant molar ratio Gal/Glc/GalA/GlcA of 2/4/1/2 determined for GY785 DR after monosaccharide analyses was in agreement with the molar ratio of the native polysaccharide, indicating that the depolymerization process had no major impact on the polysaccharide structure. After depolymerization, it was observed that when GY785 DR was mixed with an aqueous solution of CaCl_2_, the resulting mixture rapidly gelled (data not shown). Short individual fibres obtained after depolymerization observed by AFM (Figure 2B) were indeed able to associate in the presence of Ca^2+^ and form longer ones, due to chain-chain interactions mediated by these divalent cations (Figure 2C). AFM imaging was carried out on dried highly-diluted solutions, which allow to follow the initial step of gelation mechanism, that is, first inter-chain associations before a dense network formation [32,35]. Height measurements performed on GY785 DR fibres indicated an increase in fibre diameter from 0.55 ± 0.12 nm to 2.0 ± 0.4 nm, before and after calcium addition, respectively (Figure 3). By taking into account the diameter of a single monomolecular chain of ~0.5 nm reported in some previous studies [32,36] and the measured diameters, it comes out that approximately four polysaccharide chains were associated through Ca^2+^ ions.

**Figure 4:**
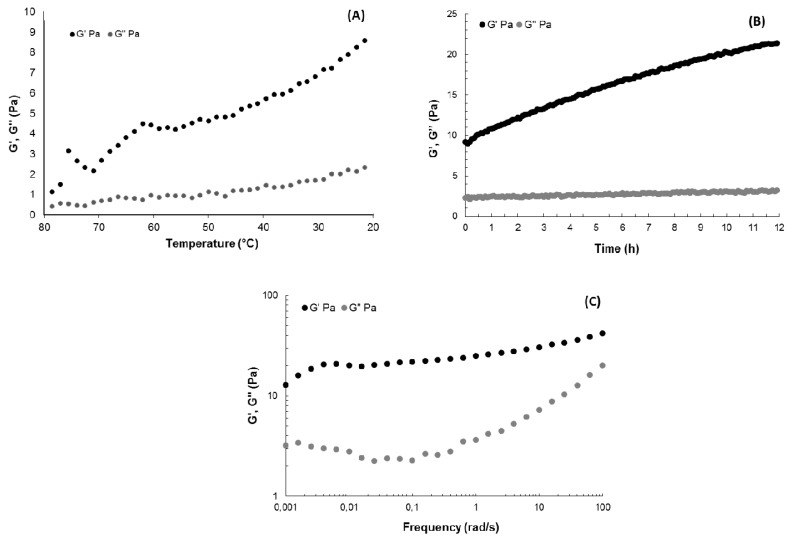
(**A**) Temperature evolution of G’ and G” (0.5% strain, 1 rad/s), (**B**) time evolution of G’ and G” at 20 °C (0.5% strain, 1 rad/s) and (**C**) mechanical spectra showing the frequency-dependence of G’ and G” at 0.5% strain at 20 °C for GY785 DR/CaCl_2_ system (GY785 DR and CaCl_2_ aqueous solutions prepared both at 40 mg/mL).

Gel formation behaviour of GY785 DR in the presence of Ca^2+^ was also investigated by rheological measurements in dynamic oscillatory mode. Evolution of storage modulus G’ and loss modulus G” during the decrease in temperature from 80 °C to 20 °C was shown on Figure 4A, whereas variation of G’ and G” as a function of time at 20 °C was presented on Figure 4B. G’ was over G” for all the cooling step and G”/G’ (tan delta values) decreased with temperature, evidencing the structuration of the system due to the complexation of Ca^2+^, in agreement with the visual observation of gelation in a glass tube. G’ evolved slightly during the 12 h kinetics, from around 9 Pa to reach 20 Pa (Figure 4B). Mechanical spectra obtained at the end of the kinetics at 20 °C showed that G’ was higher than G” over all the frequency range (Figure 4C). Over 4 × 10^−3^ rad/s, the G’ frequency dependence was very low, evidencing a structuration of the system in the plateau zone. However, at the lower frequencies, the slope of G’ increased, suggesting a flow behaviour, close to the one of an entangled semi-dilute solution, although no cross-over between G’ and G” was reached in this frequency range. The shape of G” indicated a relaxation time distribution of the macromolecular chains in the system. Calcium ions then induced a weak gel structuration, even if at lower frequencies, the system can flow, highlighting the weakness of the structuration. Such a weak gel behaviour was also observed in the absence of calcium for xanthan gum, another bacterial polysaccharide, for which the moduli presented a constant slope at high frequencies and a cross-over of G’ and G” at the lowest ones [37]. In the case of GY785 EPS, the presence of only three uronic acids per repeating unit and side chains in the EPS structure may limit the strong junction zone formation via Ca^2+^, which results in a particularly weak gel even at high polysaccharide and cross-linking agent concentrations.

### 2.2. Microcarrier Formation using Capillary Microfluidics 

**Figure 5:**
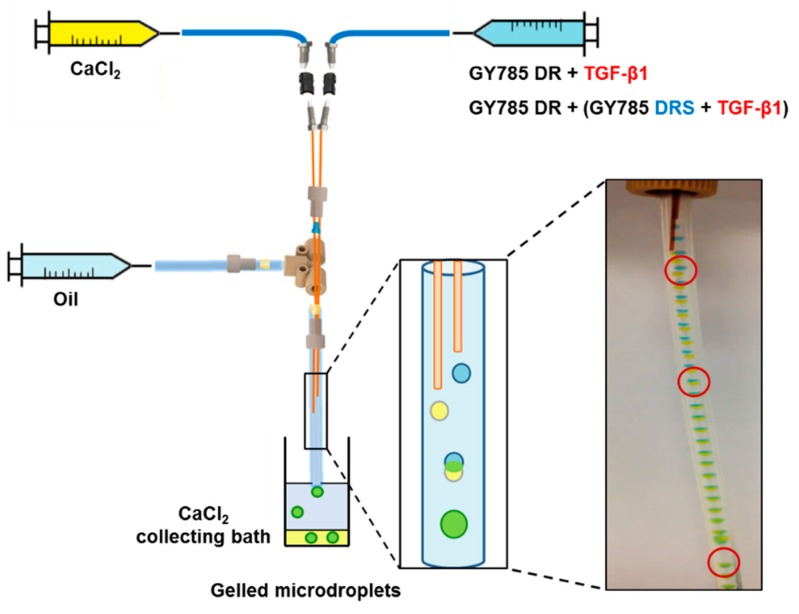
Scheme of the capillary microfluidic device with a microdroplet coalescence approach designed to generate the single GY785 DR/TGF-β1 and the double GY785 DR/DRS/TGF-β1 microcarriers.

In the following step, GY785 DR (40 mg/mL) was structured into gelled microparticles in the presence of Ca^2+^ (40 mg/mL) ions for TGF-β1 encapsulation using a capillary microfluidic technique. For the purpose of the present study, a capillary microfluidic device built from commercial chromatography components was designed (Figure 5). Two dispersed phases composed of GY785 DR and CaCl_2_ aqueous solutions were delivered through parallel silica tubes inserted into chromatography tee fitting. The continuous oil phase was delivered perpendicularly to the tee fitting. The co-flow geometry was finally obtained. As assessed by dynamic oscillatory measurements, GY785 DR formed a weak gel in the presence of Ca^2+^ ions (Figure 4). Therefore, to initiate the polysaccharide gelation directly in the capillary microfluidic device, the height of the silica tubes was adjusted to induce the coalescence of two microdroplets containing GY785 DR and CaCl_2_ solutions. To visualize the coalescence, food dyes were firstly added to the dispersed phases during the first run of experiments. After coalescence, the resulting microdroplet had changed colour to green, indicating the efficient mixing of yellow (CaCl_2_) and blue (GY785 DR) microdroplets (Figure 5). For TGF-β1 encapsulation within GY785 DR gelled microparticles (single microcarrier, GY785 DR/TGF-β1), no dyes were further used and the flow rate ratio of CaCl_2_/GY785 DR phases was set at ~27 to decrease the microdroplet diameter. At the end of experiment, the microcarriers were recovered in CaCl_2_ collecting bath. The growth factor was mixed with the GY785 DR aqueous solution before its injection into the device. In order to additionally protect the growth factor, the second microcarrier was developed. TGF-β1 was firstly incubated with LMW derivative (31,000 g/mol) of high sulphur content (14 wt%), namely GY785 DRS, for 1 h at 37 °C. The resulting GY785 DRS/TGF-β1 complex was then mixed with a GY785 DR aqueous solution prior to injection into the capillary microfluidic device to obtain the double microcarrier, GY785 DR/DRS/TGF-β1. In a previous study, GY785 DRS was shown to stimulate the hASC chondrogenic differentiation in vitro in the presence of TGF-β1, probably through non-covalent interactions with the growth factor [28]. Upon incubation, positively charged TGF-β1 (pI ~ 8.8) and negatively charged GY785 DRS were recently shown to spontaneously co-assemble and form nanoparticles [38]. This tight association between both entities may preserve the growth factor from proteolytic degradation, thus enhancing its bioactivity and its lifespan. 

**Figure 6:**
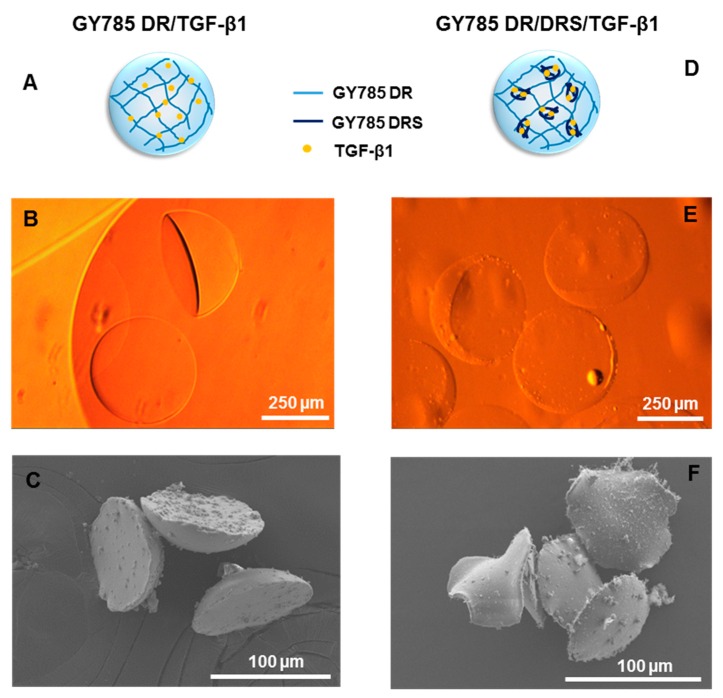
Single GY785 DR/TGF-β1 microcarrier: (**A**) schematic representation of the microparticle section, (**B**) phase contrast optical image and (**C**) SEM image. Double GY785 DR/DRS/TGF-β1 microcarrier: (**D**) schematic representation of the microparticle section, (**E**) phase contrast optical image and (**F**) SEM image.

Optical microscopy and scanning electron microscopy (SEM) observations revealed a particular morphology of the microcarriers formed (Figure 6). For both systems schematically represented on Figure 6A,D, hemi-spherical microparticles of 375 ± 10 µm (*N* = 20) in section for a single GY785 DR/TGF-β1 microcarrier and 365 ± 7 µm (*N* = 20) for a double GY785 DR/DRS/TGF-β1 microcarrier were obtained (Figure 6B,E). SEM observations performed on supercritically-dried samples, which preparation preserves the morphology of, even if the microparticle size is considerably decreased, revealed that both microcarriers display similar morphologies with GY785 DR/DRS/TGF-β1 microparticles remaining slightly thinner (Figure 6C,F). The particular hemi-spherical shape obtained can result from the fact that the cross-linking reaction initiated by the microdroplet coalescence was not complete when the droplet fell into the CaCl_2_ collecting bath. The impact force led to the formation of a flat bottom of the droplet, while the upper half retained a spherical shape. Hu et al. [39] have shown that by adjusting the height ratio of the oil/aqueous layer in collecting bath, different morphologies of alginate microgels can be obtained, including hemi-spherical microparticles. 

### 2.3. In vitro TGF-β1 Release from the Microcarriers and TGF-β1 Bioactivity Assay

**Figure 7:**
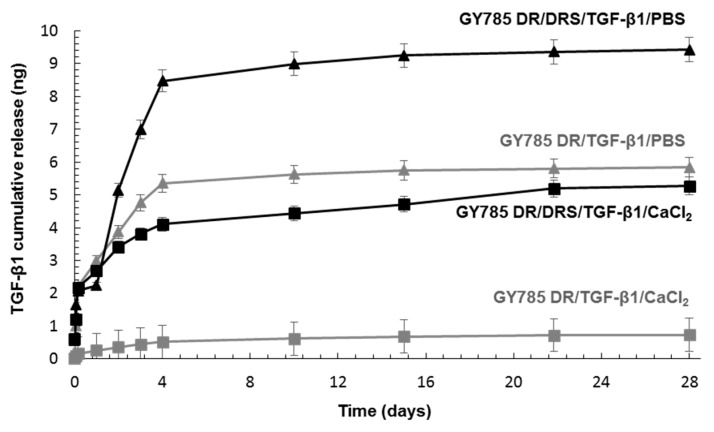
TGF-β1 cumulative release from the single GY785 DR/TGF-β1 and double GY785 DR/DRS/TGF-β1 microcarriers upon incubation with a CaCl_2_ aqueous solution at 4 °C and PBS buffer pH 7.4 at 37 °C for 28 days.

The TGF-β1 release was studied under in vitro model conditions by incubating microsystems in either CaCl_2_ aqueous solution at 4 °C or PBS buffer pH 7.4 at 37 °C both containing 0.1% HSA. The growth factor release was followed for 28 days (1 h, 4 h, 24 h, 48 h, 72 h, 96 h, 240 h, 360 h, 524 h, 672 h). By taking into account the run time for the microparticle production (20 min), the flow rates of the dispersed phases and the growth factor concentration, it can be roughly estimated that 473 ng of TGF-β1 were encapsulated per tube used for each release experiment. The growth factor release was firstly assessed in a cross-linking agent solution, CaCl_2_ at 4 °C, which models the conditions that could be applied for the microparticle storage. Upon incubation, less than 1 ng of TGF-β1 was released from the single GY785 DR/TGF-β1 system, while up to 5 ng of TGF-β1 were liberated from the double GY785 DR/DRS/TGF-β1 system, with the burst release of 2 ng during the first 4 h of incubation (Figure 7). It can be thought that the presence of GY785 DRS/TGF-β1 complex inside GY785 DR microparticles disturbs the gel formation through Ca^2+^ cross-linking, which results in lower network density and higher mesh sizes in comparison to the network formed by the single microcarrier. This network structure may favour higher growth factor diffusion from the double microcarrier. By taking into account the amount of growth factor loaded and the amount released from the microparticles, it can be estimated that 0.2% and 1.1% of initially loaded TGF-β1 were released during 28 days of storage from the single and double systems, respectively. When incubated in PBS buffer pH 7.4 at 37 °C, which models the conditions used for cell culture, the initial TGF-β1 burst release of 2 ng during the first 4 h of incubation with the buffer was observed for the single GY785 DR/TGF-β1 and the double GY785 DR/DRS/TGF-β1 systems (Figure 7). A progressive increase in released growth factor was then noticed with the rate of ~1 ng/day and ~2 ng/day from the single and double microcarriers up to 4 days, respectively. Afterwards, the amount of growth factor released increased slightly up to 28 days to reach 5.8 ng and 9.4 ng of TGF-β1 liberated from the single and the double systems, respectively. Higher TGF-β1 amount released in PBS buffer compared to CaCl_2_ aqueous solution may be due to a progressive disassembly of the microparticles caused by chelating properties of the buffer. The release study has revealed that the majority of growth factor initially loaded in the microparticles was retained in both microcarriers, whatever the buffer used. As recently shown by AFM experiments, although TGF-β1 strongly interacts with the highly sulphated derivative, GY785 DRS and tends to form the nanoassemblies, the protein affinity toward a slightly sulphated derivative, GY785 DR, was also demonstrated [38]. Thus, the low growth factor release results most likely from these non-covalent polysaccharide-growth factor interactions, which efficiently retain the growth factor inside the microparticles. 

**Figure 8:**
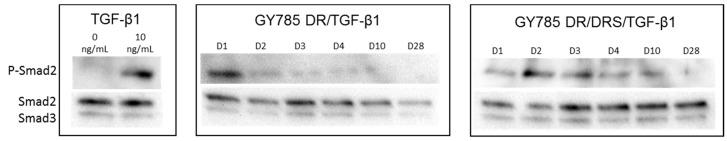
The bioactivity study of TGF-β1 released from the single GY785 DR/TGF-β1 and double GY785 DR/DRS/TGF-β1 microcarriers upon incubation with PBS buffer pH 7.4 at 37 °C for 28 days. Smad2, Smad3 and P-Smad2 were detected by Western Blot using specific antibodies, as described in the materials and methods section. Native TGF-β1 (10 ng/mL) was used as internal control.

The bioactivity of the growth factor released from the microparticles incubated with PBS buffer was then assessed. Among its various biological effects, TGF-β1 is known to be a potent inducer of the MSC chondrogenic differentiation through activation of the SMAD signalling pathways [40]. Therefore, the phosphorylation of Smad2/3 intracellular signalling proteins was investigated after stimulation of hASC with supernatants obtained after incubation of single and double microcarriers in PBS buffer for 28 days (Figure 8). In the case of TGF-β1 released from the single GY785 DR/TGF-β1 microcarriers, the phosphorylation of Smad2/3 was difficult to detect, except at day 1. This might be due to too low growth factor concentration in the supernatants rather than to TGF-β1 inactivity. However, in the double GY785 DR/DRS/TGF-β1 microcarriers, TGF-β1 was clearly shown to maintain its biological activity, as evidenced by a robust activation of Smad2 phosphorylation, particularly for supernatants collected from day 1 to day 10.

Different growth factor release ratios were reported under in vitro conditions depending on the affinity of the signalling protein toward the matrix used for its encapsulation. Very rapid TGF-β3 release from alginate microspheres was observed upon in vitro conditions, where the totality of the encapsulated growth factor was liberated upon 2 days [19]. Nearly 45% of the total TGF-β1 initially loaded were released in vitro upon 7 days from chitosan microspheres [41]. In another study, less than 20% of BMP-2 were released from heparin microparticles, indicating that the majority of the growth factor was retained in the matrix [42]. However, even if the growth factor remained bound to the polysaccharide matrix, it displayed similar biological activities compared to the released one. BMP-2 retained inside heparin microparticles induced a functional cellular response, which suggests that heparin may enhance BMP-2 cell signalling by facilitating the formation of growth-factor—receptor complexes on the cell surface or by prolonging its lifespan in culture [42]. In vitro and in vivo studies performed on an alginate/alginate sulphate scaffold with affinity bound TGF-β1 indicated that the efficient capture and sustained presentation of the growth factor enabled the induction of signalling pathways leading to chondrogenesis, up to the appearance of committed chondrocytes specific of the hyaline cartilage type [20]. Therefore, systems acting as reservoirs of growth factors, which provide a protective microenvironment in the same manner as GAG in the ECM are particularly interesting for tissue regeneration requiring a long period of time to allow the cells to migrate to the site of injury, proliferate and differentiate into appropriate cell phenotype.

## 3. Materials and Methods 

### 3.1. Production of the Native GY785 EPS

The production of the GY785 EPS was previously described [26]. Here, *Alteromonas infernus* was cultured in Zobell medium composed of tryptone (5 g/L), yeast extract (1 g/L) and aquarium salts (33.3 g/L) at 25 °C and pH 7.4 in a 30 L fermenter (Techfors 30 L INFORS, Switzerland) with 30 g/L of glucose, as a carbohydrate source. After 48 h of fermentation, the culture medium was centrifuged (9000 g, 45 min) and the supernatant containing soluble EPS was ultrafiltrated on a 100 kDa cut-off membrane and freeze-dried.

### 3.2. Preparation of GY785 EPS Derivatives: GY785 DR and GY785 DRS 

GY785 DR was obtained by a free-radical depolymerization process, as previously described [43,44]. Briefly, 2.5 g of the native EPS were solubilized in water (350 mL) and an aqueous solution of hydrogen peroxide was added dropwise to depolymerize the EPS in the presence of copper (II) acetate used as catalyst. After overnight reduction using sodium borohydride and purification on Chelex^®^ 20 resin, the solution containing EPS was ultrafiltrated on a 10 kDa cut-off membrane and freeze-dried. To obtain homogeneous fractions of GY785 DR with low polydispersity, a predominant population of polysaccharide chains was selected by a gel filtration chromatography on either Superdex^®^ 30 (GY785 DR of ~20,000 g/mol) or Sephacryl S-100 HR (GY785 DR of ~200,000 g/mol) (GE Healthcare Life Sciences), using an AKTA FPLC system coupled with a refractometric detector (Gilson^®^). Samples eluted with water were pooled and freeze-dried.

Highly sulphated GY785 DRS was obtained by a chemical sulphation of GY785 DR of ~20,000 g/mol, as described earlier [43,44]. GY785 DR (50 mg) in its pyridinium salt form was firstly solubilized in anhydrous DMF (100 mL) for 2 h at 45 °C under continuous stirring and then sulphated for the next 2 h at 45 °C in the presence of SO_3_∙Py (250 mg). The final aqueous solution (pH 7) was dialyzed against water for 3 days before being freeze-dried.

### 3.3. Characterization of GY785 EPS Derivatives

#### 3.3.1. Sugar Composition 

Monosaccharide composition was determined according to Kamerling et al. [45] method, modified by Montreuil et al. [46] Native GY785 EPS and its derivatives were hydrolysed using MeOH/HCl for 4 h at 100 °C. *Myo*-inositol was used as internal standard. The methyl glycosides thus obtained were then converted to trimethylsilyl derivatives using *N*,*O*-bis(trimethylsilyl)trifluoroacetamide and trimethylchlorosilane (BSTFA:TMCS) 99:1 (Merck). Gas chromatography (GC-FID, Agilent Technologies 6890N) was used for separation and quantification of the per-*O*-trimethylsilyl methyl glycosides formed. 

#### 3.3.2. Molecular Weight

High-performance size-exclusion chromatography (HPSEC, Prominence Shimadzu, Kyoto, Japan) coupled with a multiangle light scattering (MALS, Dawn Heleos-II, Wyatt Technology, Santa Barbara, CA, USA) and a differential refractive index (RI) (Optilab Wyatt technology, Santa Barbara, CA, USA) detectors was applied to determine the weight-average molecular weight of the native GY785 EPS and its derivatives. 

#### 3.3.3. Sulphate Content

High-Performance Anion-Exchange Chromatography (HPAEC) was used to quantify the linked ester sulphate group content in the native GY785 EPS and its derivatives, as previously described by Chopin et al. [44]. HPAEC analyses were carried out with a Dionex DX-500 ion chromatographic instrument controlled using Chromeleon^®^ software. 

### 3.4. Atomic Force Microscopy (AFM): Sample Preparation and Imaging 

Native GY785 EPS and its derivative, GY785 DR (240,000 g/mol), were firstly solubilized overnight at 1 mg/mL in water before being diluted at 5 µg/mL. CaCl_2_ (Sigma) aqueous solution at 10 mg/mL was mixed (1:1, *v*/*v*) with GY785 DR aqueous solution at 1 mg/mL. The remaining mixture was then diluted at 5 µg/mL in water. 5 µL of each diluted solution were deposited onto freshly cleaved mica surface and dried at under ambient conditions. AFM images were recorded using a NanoWizard^®^ Atomic Force Microscope (JPK, Berlin, Germany) in intermittent contact mode at room temperature. A standard rectangular cantilever (Nanosensors NCL-W) with a free resonance frequency of 165 kHz and a spring constant of 40 N/m was used. The AFM tips with a radius curvature of ~10 nm were cleaned by UV-ozone treatment prior to AFM observations. Each sample was imaged on three different zones. JPK Data Processing software (JPK, Germany) was used for image processing and height measurements.

### 3.5. Gelling Properties of the Native GY785 EPS and GY785 DR

Gelling ability of the native GY785 EPS and its derivative, GY785 DR (240,000 g/mol) was assessed by mixing (1:1, *v*/*v*), in a glass tube, an aqueous solution of the polysaccharide at 40 mg/mL (pH 7) with an aqueous solution of CaCl_2_ at 40 mg/mL. To evaluate gelation, tubes were immediately reversed. 

### 3.6. Rheological Measurements

GY785 DR (240,000 g/mol) and CaCl_2_ aqueous solutions prepared both at 40 mg/mL were mixed (1:1, *v*/*v*) at 80 °C and deposited on the preheated rheometer plate maintained at the temperature of solution. The temperature was then decreased to 20 °C at 1 °C/min. Time sweep oscillatory measurements were performed at a frequency of 1 rad/s for a strain amplitude of 0.5% using a controlled stress rheometer (AR2000, TA Instruments) equipped with a Peltier temperature controller and with a cone-plate device (40 mm diameter, 4° angle, 98 µm air gap). Frequency sweep oscillatory measurements (from 0.001 rad/s to 100 rad/s, 0.5% strain, 20 °C) were performed at the end of 12 h kinetics. At the end of experiments, a strain sweep test was performed in order to check that measurements had been done within the linearity limits of the viscoelastic behaviour. 

### 3.7. Microcarrier Formation Using Capillary Microfluidics 

Single microcarriers, GY785 DR/TGF-β1, were prepared by mixing GY785 DR (240,000 g/mol) aqueous solution (final concentration of 40 mg/mL) with TGF-β1 (CHO cells, PeproTech, Neuilly-sur-Seine, France) (final concentration of 50 µg/mL). To prepare double microcarriers, GY785 DR/DRS/TGF-β1, GY785 DRS (31,000 g/mol) and TGF-β1 aqueous solutions at 1 mg/mL were firstly mixed and incubated for 1 h at 37 °C under gentle stirring. The resulting GY785 DRS/TGF-β1 complex (GY785 DRS and TGF-β1 final concentrations of 0.05 mg/mL and 50 µg/mL, respectively) was added to GY785 DR aqueous solution (final concentration of 40 mg/mL). To structure GY785 DR (240,000 g/mol) into gelled microparticles, a capillary microfluidic set-up was developed. A microfluidic device with a double co-axial flow focusing geometry was used. Two dispersed phases: CaCl_2_ aqueous solution at 40 mg/mL and either GY785 DR/TGF-β1 or GY785 DR/DRS/TGF-β1 aqueous solutions, were delivered through two parallel silica tubes (interior diameter (ID) 75 µm and outside diameter (OD) 360 µm) (IDEX, US) inserted into chromatography tee fitting (Cluzeau Info Labo, France). The height between silica tubes was shifted (0.5 cm) to induce the coalescence of microdroplets formed. To visualize the coalescence, food dyes were firstly added to the dispersed phases before TGF-β1 encapsulation. The continuous phase, sunflower seed oil (Fluka), was delivered through a Fluorinated Ethylene Propylene (FEP) 1/16” tube (ID 750 µm, OD 1.57 mm) (Cluzeau Info Labo, France) perpendicularly to the tee fitting. The two dispersed phases co-flowed in a FEP 1/16” tube (ID 750 µm, OD 1.57 mm) that contains the continuous phase. The final double co-flow geometry was obtained. Digitally controlled syringe pumps (Harvard Apparatus PHD 2000, France) delivered all liquid phases to the device. The following flow rates of the dispersed phases: CaCl_2_ at 12 µL/min and EPS/TGF-β1 solutions at 0.45 µL/min and of the continuous phase: oil at 500 µL/min were applied. The device was run for 20 min per tube. The microparticle suspension recovered in 20 mg/mL CaCl_2_ collecting bath was washed three times with the same solution and used immediately for release experiments. 

### 3.8. Optical and Scanning Electron Microscopy (SEM) Observations 

Phase contrast images of gelled microparticles were captured with an Olympus IX51 inverse microscope (Olympus, France) equipped with a digital camera (Sony, SCD-SX90). The size distribution of microparticles produced were analysed using the ImageJ freeware v135c. SEM observations on supercritically-dried samples prepared as previously described [31] were made using a Jeol 6400F microscope operating at 3 kV after gold/palladium sample coating.

### 3.9. In Vitro TGF-β1 Release from the Microcarriers 

For in vitro release study, the suspensions of the single and double microcarriers (350 µL) were incubated in either 10 mM PBS at pH 7.4 containing 0.1% Human Serum Albumin (HSA, Sigma) (350 µL) at 37 °C under gentle shaking or CaCl_2_ at 20 mg/mL with 0.1% HSA (350 µL) at 4 °C for 28 days. For different incubation times (1 h, 4 h, 24 h, 48 h, 72 h, 96 h, 240 h, 360 h, 524 h, 672 h), 350 µL of the supernatant was removed after centrifugation step (10 min, 2000 g) and replaced by fresh buffer. The concentration of released TGF-β1 from the microcarriers was determined using ELISA Duoset^®^ assay kit (RnD Systems).

### 3.10. TGF-β1 Bioactivity Assay after Release

Human adipose-derived stromal cells (hASC) were cultured in Dulbecco’s Modified Eagle Medium (DMEM, ThermoFisher Scientific) supplemented with 10% Foetal Bovine Serum (FBS, Dominique Dutscher) and 1% Penicillin/Streptomycin (P/S, ThermoFisher Scientific) at 37 °C and 5% CO_2_, as previously described [23,28]. Cells were then seeded on six-well plates at 10,000 cells/cm^2^ and proliferated until reaching 80% confluence. They were then serum starved for 24 h with initial medium prior to stimulation with TGF-β1 released from microcarriers during their incubation in PBS buffer pH 7.4 at 37 °C for 28 days. Growth factor standards and supernatants (24 h, 48 h, 72 h, 96 h, 240 h, 672 h) were incubated with hASC for 1 h at 37 °C. The medium was then removed and proteins were immediately extracted on ice using homemade RIPA buffer and assayed with the BCA™ Protein Assay Kit (ThermoFisher Scientific). For Western Blot experiments, after migration on AnykD Criterion™ TGX™ precast gels (Bio-Rad), proteins were transferred onto 0.2 µm PVDF membranes (Bio-Rad) using the Transblot^®^ Turbo™ transfer system (Bio-Rad). Total Smad 2/3 and Phosphorylated-Smad 2 (P-Smad2) were analysed using specific antibodies (Cell Signalling) for cells stimulated with TGF-β1. All primary antibodies were revealed using a horseradish peroxidase-coupled secondary antibody (Cell Signalling) and the SuperSignal™ West Dura substrate (ThermoFisher Scientific) on the ChemiDoc™ MP Imaging System (Bio-Rad) and the Image Lab software.

## 4. Conclusions

In the present study, an atypical EPS from marine origin, namely GY785 EPS was structured into microparticles for long-term TGF-β1 protection. Both functional, that is, physico-chemical (gelling) and biological (GAG-like) properties of this EPS were exploited to create functional microcarriers, which could further be used for cartilage regeneration. To preserve its bioactivity, TGF-β1 was encapsulated in mild conditions inside gelled microparticles formed using a capillary microfluidic technique. To initiate the polysaccharide cross-linking through physical gelation mediated by Ca^2+^ ions directly into the microfluidic device, the microdroplet coalescence approach was developed. In the single GY785 DR/TGF-β1 microcarrier, the growth factor was directly encapsulated within the polysaccharide microparticule, while in the double GY785 DR/DRS/TGF-β1 microcarrier, the growth factor was firstly complexed with the highly sulphated derivative, shown in a previous study to enhance hASC chondrogenic differentiation. The resulting GY785 DRS/TGF-β1 complex was then incorporated within the microparticles during their formation. The release kinetics differed slightly between both microcarriers in two different buffers, with higher TGF-β1 release from the double microcarrier. The presence of GY785 DRS/TGF-β1 nanoassemblies probably disturbs gelation conducting to a polysaccharide network of lower density and higher mesh size. The bioactivity study performed has shown that the growth factor activity was particularly maintained in the presence of highly sulphated derivative. In both microcarriers, the majority of the encapsulated growth factor was however retained for 28 days. GY785 EPS based microcarriers may therefore constitute TGF-β1 reservoirs that provide long time protection of the growth factor. This feature could be particularly interesting for various tissue engineering applications, including cartilage repair, where the presence of growth factors is required for extended times to promote tissue regeneration.
